# Antimicrobial and cytotoxic activity of green synthesis silver nanoparticles targeting skin and soft tissue infectious agents

**DOI:** 10.1038/s41598-021-94012-y

**Published:** 2021-07-15

**Authors:** Javier Mussin, Viviana Robles-Botero, Rocío Casañas-Pimentel, Florencia Rojas, Letizia Angiolella, Eduardo San Martín-Martínez, Gustavo Giusiano

**Affiliations:** 1 Mycology Department, Instituto de Medicina Regional, Universidad Nacional del Nordeste, Consejo Nacional de Investigaciones Científicas y Tecnológicas (CONICET), Av. Las Heras 727, 3500 Resistencia, Chaco, Argentina; 2 Centro de Investigación en Ciencia Aplicada y Tecnología Avanzada, CONACYT - Instituto Politécnico Nacional, Mexico City, Mexico; 3 Department of Public Health and Infectious Diseases, University of Rome “Sapienza”, Rome, Italy; 4 Centro de Investigación en Ciencia Aplicada y Tecnología Avanzada - Legaria, Instituto Politécnico Nacional, Mexico City, Mexico

**Keywords:** Skin diseases, Nanoparticles, Fungi

## Abstract

Combining traditional medicine with nanotechnology therefore opens the door to innovative strategies for treating skin and soft tissue infections (SSTIs) and also contributes to the fight against the rise of antimicrobial resistance. *Acanthospermum australe* (Loefl.) Kuntze is a medicinal plant used by indigenous peoples in northeastern Argentina to treat SSTIs. Spherical and stable silver nanoparticles (AgNPs) of 14 ± 2 nm were synthesized from the aqueous extract of *A. australe* and silver nitrate. The antimicrobial activity against main species causing SSTIs and cytotoxicity on peripheral blood mononuclear cells of AgNP solution and its synthesis components were evaluated. Compared to its synthesis components, AgNP solution showed greater antimicrobial activity and lower cytotoxicity. The antimicrobial activity of AgNPs was due to the silver and not to the metabolites of the aqueous extract present on the surface of the nanoparticles. The plant extract played an important role in the formation of stable AgNPs and acted as a modulator of cytotoxic and immune responses.

## Introduction

The skin is the largest organ of the human body and forms an integral part of the immune system, serving as the first line of defense against microbial infections. Skin and soft tissue infections (SSTIs) are a common reason for consultations in primary health care centers. They are clinical entities with varying manifestations, etiologies, and severities, ranging from mild to life-threatening infections. They involve microbial invasion of the skin layers and soft tissues, followed by a process that leads to clinical effects as a result of the interaction between microorganisms and host defenses. Furthermore, the pathophysiology of SSTIs is the result of a complex interplay of physiological, immunological, and environmental effects, including microorganism, inflammation, oxidative stress, and impaired healing. In immunocompetent hosts, SSTIs can be caused by various microorganisms, mainly bacteria and fungi^1–3^.

The most common bacteria implicated in SSTIs are *Streptococcus pyogenes*, *Pseudomonas aeruginosa*, and *Staphylococcus aureus*, including methicillin-resistant *S. aureus* strains. Superficial fungal infections are mainly caused by dermatophytes and yeasts. Dermatophytes are a group of fungi capable of invading keratinized structures (skin, hair, and nails), mediated by both keratinases and proteases. The main dermatophytes that cause human dermatomycosis belong to the genera *Trichophyton*, *Microsporum*, and *Epidermophyton*, while *Candida* spp. and *Malassezia* spp. are the most common yeasts. These yeasts are recognized members of the human skin microbiota, but under certain conditions they can change their status and act as pathogens^2^. In addition, *Malassezia* spp. (lipophilic yeasts) are the most abundant eukaryotes, accounting for approximately 50–80% of the total skin mycobiome^4,5^.

Due to the increase in strains resistant to antimicrobial agents, skin infections are increasingly difficult to treat. Infections caused by drug-resistant microorganisms are associated with high morbidity and mortality and consequently higher healthcare costs^6^. Therefore, there is a need to find/develop compounds with antimicrobial properties that are also cost effective.

Nanotechnology is an important modern field of research that deals with the synthesis and manipulation of matter at the nanometer scale (1 nm = 10^–9^ m) and the study of its properties and applications. Nanomedicine has emerged as an interdisciplinary science that combines knowledge from physics, chemistry, biology, and medicine to treat human diseases. Currently, the development of nanoparticle-based products is growing, many of which are already on the market^7,8^. Noble metal nanoparticles have demonstrated unique and significantly different physical and chemical properties than their macroscopic counterparts. As nanoparticles decrease in size, their surface to volume ratio, dispersion, and antimicrobial activity increase^7,9–13^. Silver is a noble metal with high antimicrobial activity and low toxicity in animal cells and has a long history in traditional medicine^11^. Various silver compounds have been used clinically to reduce skin infections (e.g. silver sulfadiazine). Currently, there is a growing interest in the use of silver nanoparticles (AgNPs) for the treatment of SSTIs due to their improved properties^8,11^.

On the other hand, nanoparticles are usually synthesized through a variety of physical and chemical processes that are costly and pollute the environment. For this reason, plant-mediated synthesis has emerged as a green synthesis method that utilizes plants as a source of metabolites, mainly phenolic compounds, for the synthesis of nanoparticles as an environmentally friendly technology^10,12^.

Plants have been used as medicine by various cultures throughout history. Their myriad uses have been documented and passed down through generations. They are an important source of phenolic compounds, mainly flavonoids, many of which have antimicrobial, anti-inflammatory and antioxidant properties that enhance the treatment of SSTIs. *Acanthospermum australe* (Loefl.) Kuntze, commonly known as "tapekué" in Argentina, is a medicinal plant used by indigenous peoples in northeastern Argentina to disinfect wounds and skin ulcers, among other uses^3,14,15^. In addition, silver is used in traditional Indian medicine (Ayurveda) as a therapeutic agent to treat various infections^16^. The combination of traditional medicine with nanotechnology therefore opens the door to innovative strategies for the treatment of SSTIs and also contributes to the fight against the increase in antimicrobial resistance.

The objectives of this study were (a) to synthesize and characterize AgNPs using an aqueous extract of *A. australe* (TAE), (b) to analyze the total phenolic and flavonoid content of the extract, (c) to evaluate the antioxidant activity, and (d) to determine and compare the cytotoxicity and antimicrobial activity of the AgNP solution and its synthesis components.

## Materials and methods

### Plant material

All methods were carried out in accordance with the recommendations established in a previous work based on the guidelines on good agricultural and collection practices for medicinal plants of the World Health Organization and European Medicines Agency^17^. The plant material was collected in accordance with relevant institutional, national and international guidelines and laws. The leaves of *A. australe* were harvested by hand and dried at room temperature protected from sunlight.

Plant material was collected in Riachuelo, Corrientes, Argentina (27°33′42.0"S 58°43′35.9"W) and taxonomically identified by Dr. Massimiliano Dematteis of the Instituto de Botánica del Nordeste, Universidad Nacional del Nordeste, Argentina. A voucher specimen has been deposited in the herbarium of the Instituto de Medicina Regional, Universidad Nacional del Nordeste, Argentina, with the number IMR-H-2017–18.

### Preparation of the aqueous extract of *A. australe* (TAE)

Exactly 50 g of the comminuted plant material was boiled with 1000 mL of sterile deionized water for 5 min and allowed to stand for 1 h at room temperature. It was then filtered using Whatman filter paper No. 1 followed by a Millipore filter (0.22 µm). The obtained aqueous extract was frozen at -70 °C, lyophilized and stored at 4 °C in the dark under sterile conditions for later use. The percentage yield of extraction (Y) was determined using the following equation:

$$Y = \left( {\frac{grams\;of\;lyophilized\;extract\;obtained}{{grams\;of\;plant\;material\;used}}} \right) \times 100$$

### Total phenolic content

The total phenolic content of TAE was analyzed spectrophotometrically using the Folin-Ciocalteu method^18^, with some modifications (Supplementary file). The results are expressed as the mean gallic acid equivalent mass in mg per g of lyophilized extract (mg GAE/g LE) ± standard deviation (SD).

### Total flavonoid content

It was determined for TAE according to Dewanto et al.^19^, with modifications (Supplementary file). Total flavonoid content was expressed as the mean quercetin equivalent mass in mg per g of lyophilized extract (mg QE/g LE) ± SD.

### Green synthesis of silver nanoparticles

In order to obtain small and stable nanoparticles, the green synthesis was carried out according to the results obtained by other authors^12,20,21^, with some modifications (Supplementary file).

### Characterization of silver nanoparticles

The synthesized AgNPs were characterized using the following techniques: (A) UV–visible spectrophotometer (Multiskan Go, Thermo Fisher, Finland) was used to measure absorbance and record optical density at wavelengths from 300 to 700 nm. (B) The shape and size of AgNPs were determined by transmission electron microscopy (TEM; JEOL, Tokyo, Japan; JEM-2100) at an accelerating voltage of 200 kV. Samples were prepared by drop coating 10 µL of AgNP solution onto carbon coated copper grids followed by air drying, forming a thin layer on the surface. (C) Energy dispersive X-ray spectroscopy (EDX) was performed to determine the elemental composition of the sample. (D) The hydrodynamic size, polydispersity index (PDI) and zeta potential of the AgNP colloid were investigated by dynamic light scattering (DLS) using a Malvern Zetasizer Nanoseries compact scattering spectrometer (Malvern, UK) at 25 °C. (E) Fourier transform infrared spectroscopy (FTIR) with a resolution of 0.2 nm at 40–4000 cm^-1^ (Nicolet 670, Madison, WI) was used to identify the possible biomolecules responsible for the reduction of silver ions and capping of the synthesized AgNPs. (F) The colloidal stability of AgNPs over time was evaluated by visual observation and UV–visible spectroscopy.

### Ferric reducing antioxidant power (FRAP) assay

The antioxidant activity of TAE, AgNO_3_ and AgNPs was determined using the FRAP assay according to Quiroz-Reyes et al.^22^ (Supplementary file). The results are expressed as the mean Trolox equivalent mass in μmol per g of lyophilized extract (μmol TE/g LE) ± SD.

### DPPH radical scavenging capacity

The antiradical capacity was determined using the DPPH (2,2-diphenyl-1-picrylhydrazyl) assay according to the methodology proposed by Molyneux^23^, with some modifications (Supplementary file). The effective concentration 50% (EC_50_), defined as the amount of antioxidant required to reduce the initial concentration of DPPH radical by 50%, was calculated. In addition, the percentage of DPPH reduction was calculated using the following equation:

$$DPPH\;inhibition \left( \% \right) = \left[ {1 - \frac{Sample\;absorbance}{{Blank\;absorbance}}} \right]*100$$

### Cytotoxicity assay

The cytotoxicity of TAE, AgNO_3_, and AgNPs was determined against peripheral blood mononuclear cells (PBMCs) as previously described^24^, with modifications (Supplementary file). Untreated cells were considered as negative control and paclitaxel 2.5 µg/mL (Sigma Aldrich, USA) as positive control. The different samples were compared with the negative control and the percent cell viability was calculated. Samples with viability percentages below 70% were considered cytotoxic according to the recommendations of ISO 10,993–5^25^.

### Microorganisms

A total of 298 microorganisms were examined: 33 *Microsporum canis*, 19 M*. gypseum*, 4 *Epidermophyton floccosum,* 31 *Trichophyton rubrum*, 22 T*. mentagrophytes*, 6 T*. tonsurans*, 40 *Malassezia furfur*, 25 M*. sympodialis*, 9 M*. globosa*, 1 M*. restricta*, 24 *C. albicans*, 21 *C. krusei,* 16 *C. tropicalis*, 11 *C. parapsilosis*, 6 *C. glabrata*, 8 *P. aeruginosa*, 8 *S. pyogenes*, and 14 *S. aureus* (including 2 methicillin-resistant strains).

All microorganisms were obtained from the culture collection of the Instituto de Medicina Regional, Universidad Nacional del Nordeste, Argentina. The reference strains *M. furfur* CBS 7019, *M. sympodialis* CBS 7222, *M. globosa* CBS 7986, *C. albicans* ATCC 90,028, *C. tropicalis* ATCC 750, *C. krusei* ATCC 6258, *C. parapsilosis* ATCC 22,019, *C. glabrata* ATCC 2001 and *S. aureus* ATCC 25,923 were included.

### In vitro inhibitory activity

#### Minimum inhibitory concentration (MIC)

To evaluate the inhibitory activity of TAE, AgNO_3_ and AgNPs, MICs were determined using the broth microdilution method in accordance with the CLSI M27 document for yeasts^26,27^, CLSI M38 for filamentous fungi^28^and CLSI M07 for bacteria^29^. Dimethyl sulfoxide was used as the solvent (final concentration ≤ 1%).

MIC was determined by visual reading of growth inhibition at three endpoints using the following numerical scale: MIC-0 as the lowest concentration capable of inhibiting 100% of microbial growth; MIC-1 as the lowest concentration capable of inhibiting ≥ 80% of microbial growth compared to the growth control; and MIC-2 as the lowest concentration capable of inhibiting ≥ 50% of microbial growth compared to the growth control. The criteria for antimicrobial activity defined in a previous work were followed^17^. These criteria specify that plant extracts are considered active when MIC-2 ≤ 1024 μg/mL and inactive at higher values, while pure compounds are considered active when MIC-2 ≤ 256 μg/mL and inactive at higher values. In this work, AgNP and AgNO_3_ solutions were considered as pure compounds and TAE as plant extract. The inhibitory controls used were: itraconazole (ITZ) (Sigma-Aldrich) for fungi, penicillin (PEN) (Sigma-Aldrich) for *S. pyogenes*, and gentamicin (GEN) (Sigma-Aldrich) for *S. aureus* and *P. aeruginosa*. For the control drugs, the endpoint values reported in the CLSI documents were used.

##### Minimum fungicidal concentration (MFC) and minimum bactericidal concentration (MBC)

The MFC and MBC of TAE, AgNO_3_ and AgNPs were determined according to the procedures established by other authors^30–33^, with modifications (Supplementary file). The MBC and MFC were defined as the lowest TAE, AgNO_3_ or AgNP concentrations at which no colonies were observed.

MFC:MIC and MBC:MIC ratios were calculated for each isolate using the MIC-0 value. By extrapolating the conventional definition used for bacterial assays, in this work a compound was considered bactericidal or fungicidal if the ratio was ≤ 4, and bacteriostatic or fungistatic if the ratio was > 4^33,34^.

All assays were performed in duplicates. Range and mode values were determined. All MIC, MFC, and MBC data were expressed as μg LE/mL for TAE and μg silver/mL for AgNO_3_ and AgNPs.

## Results

### Yield and phytochemical analysis

The process used to obtain the TAE showed a yield of 12%. On the other hand, the total phenolic and flavonoid contents of the extract were 155.9 ± 3.9 mg GAE/g LE and 20.97 ± 0.32 mg QE/g LE, respectively.

### Characterization of silver nanoparticles

**Figure 1:**
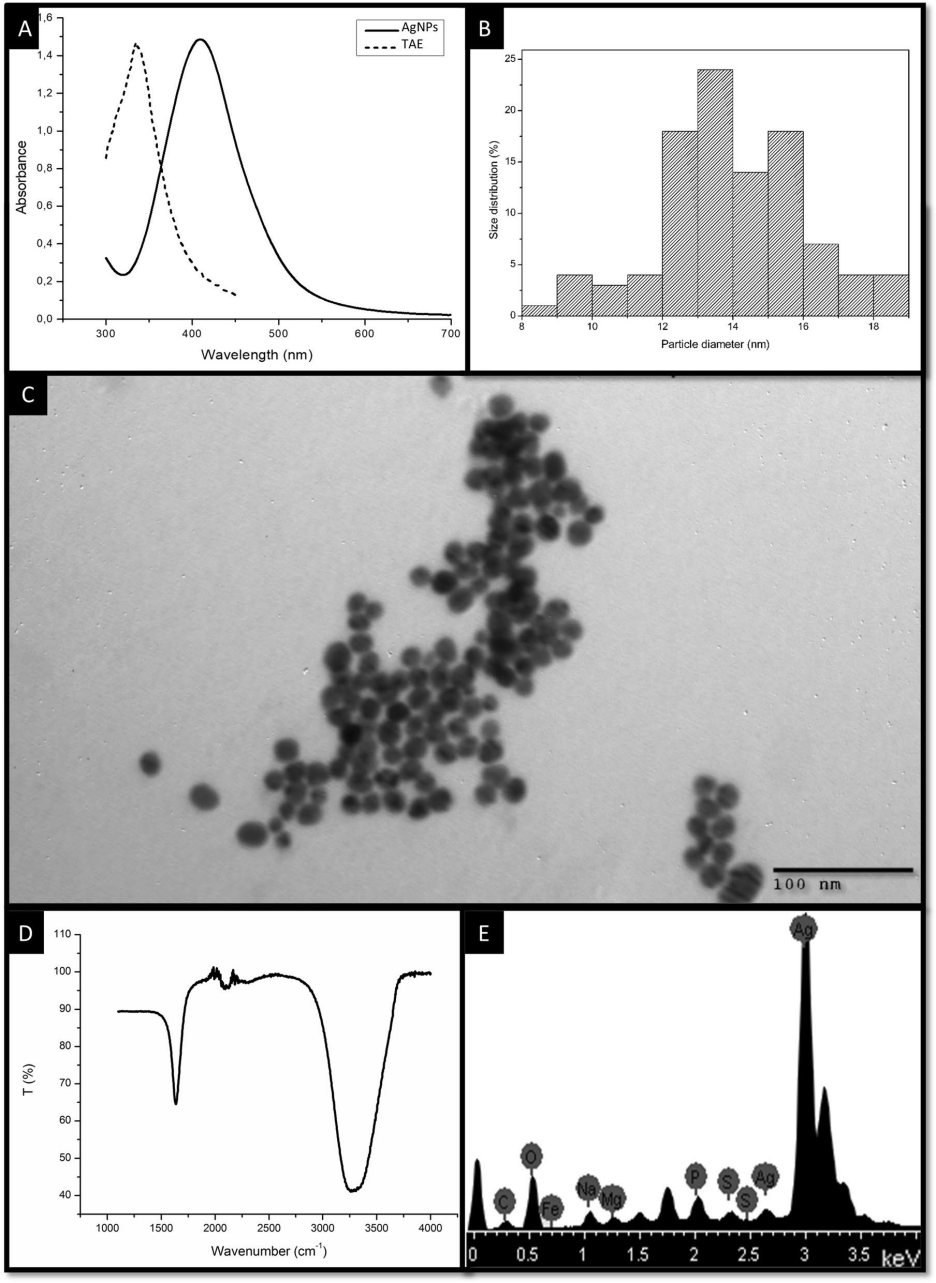
Characteristics of the synthesized AgNPs from TAE. (**A**) UV–visible spectrum. (**B**) Histogram of particle size distribution. (**C**) TEM image. (**D**) FTIR spectra. (**E**) EDX spectra.

During green synthesis, the color of the solution changed from pale yellow to dark brown within a few min, indicating the formation of AgNP. The main features of the synthesized nanoparticles are shown in Fig. 1. The UV–visible spectrum showed a maximum absorption band at 410 nm, which corresponds to the local surface plasmon resonance absorption typical of nanoparticles^35^. The TEM images showed non-agglomerated spherical nanoparticles. The sizes of at least 100 particles were measured using ImageJ software. The data were transferred to Origin software for statistical analysis, which revealed a diameter of 14 ± 2 nm. DLS analysis revealed strongly anionic nanoparticles with a PDI of 0.269, a hydrodynamic diameter of 36 ± 13 nm, and a zeta potential of -37 ± 6 mV. The FTIR spectrum of AgNPs showed peaks at about 1636, 2100 and 3300 cm^-1^. Finally, EDX analysis revealed a strong signal of elemental silver with an optical absorption band peak in the range of 3 to 4 keV, which is typical for the absorption of metallic silver nanocrystallites^35^. In addition, other elemental signals were recorded in smaller amounts.

### Antioxidant activity

The results of FRAP and DPPH assays of TAE, AgNO_3_ and AgNPs are shown in Table 1.

**Table 1:** Antioxidant activities of TAE, AgNO_3_ and AgNPs.

Sample	DPPH		FRAP*
	EC_50_†	% inhibition	
TAE	306.5 ± 1.6	77.7 ± 1.8	596 ± 10
AgNO_3_	ND	3.6 ± 1.2	0 ± 0
AgNPs	ND	35.2 ± 0.4	279 ± 1

*μmol TE/g LE; †µg LE/mL, *ND* not determined.

### Cytotoxicity assay

**Figure 2:**
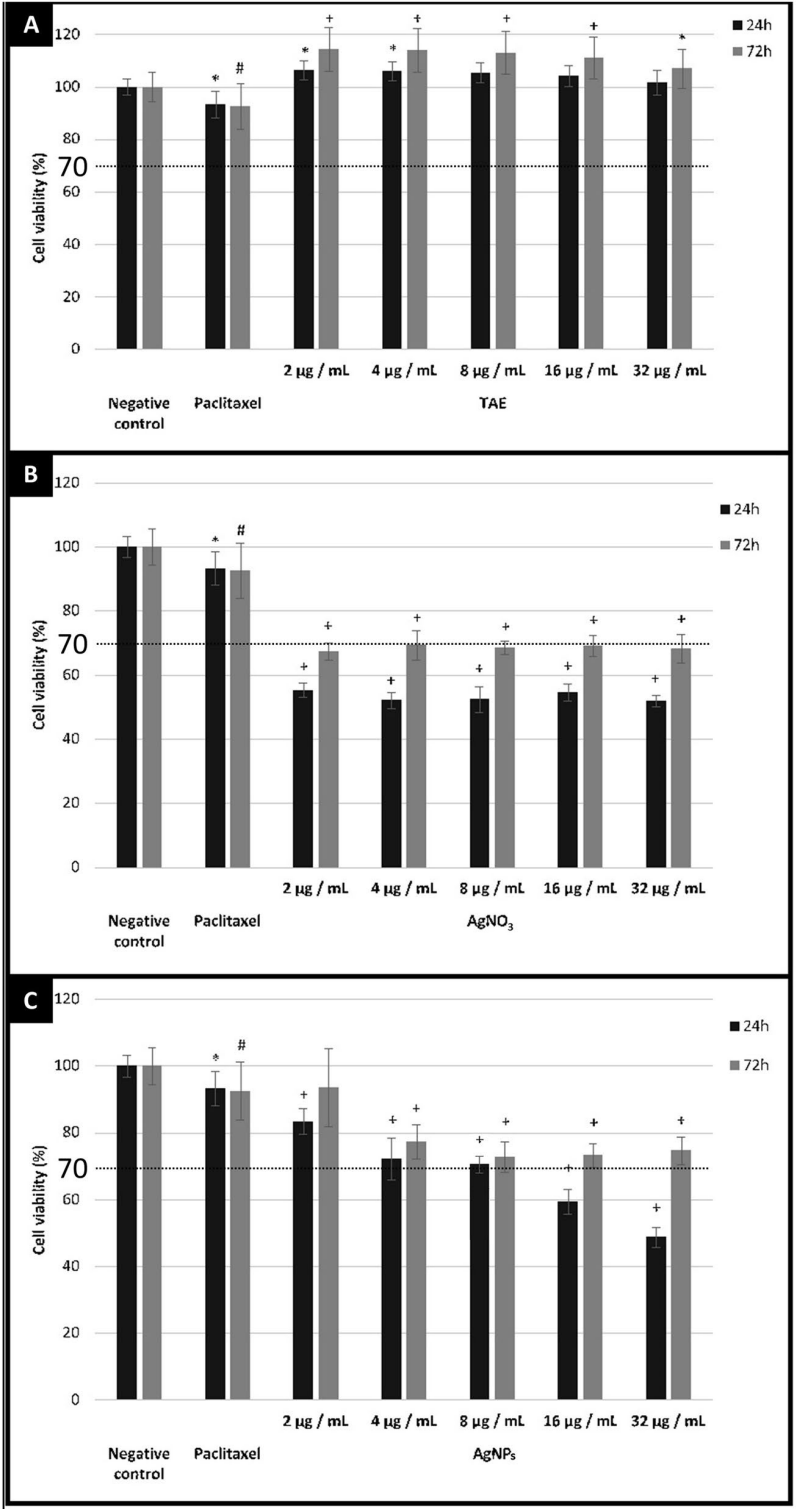
Percent cell viability after 24 and 72 h of incubation. Results are expressed as (**A**) µg LE/mL for TAE; (**B**) µg silver/mL for AgNPs; and (**C**) μg silver/mL AgNO_3_. Negative control: untreated cells. Positive control: paclitaxel (2.5 μg/mL). For each treatment and control group, the number of determinations was ≥ 12. The symbol *indicates a statistically significant difference from the negative control at *P* between 0.0137 and 0.0436; the symbol (#) indicates a statistically significant difference from the negative control at *P* between 0.0051 and 0.0067; the symbol (+) indicates a statistically significant difference from the negative control at *P* < 0.0001.

The cytotoxicity of TAE, AgNO_3_ and AgNPs was evaluated on PBMCs after 24 and 72 h incubation, and the results are shown in Fig. 2.

### In vitro inhibitory activity

Range and mode values for MIC-2, MIC-1, MIC-0, MFC, MBC, MBC:MIC and MFC:MIC obtained with TAE, AgNO_3_ and AgNPs against the tested microorganisms are shown in Table 2.

**Table 2:** Range and mode values of MIC-0, MIC-1, MIC-2, MFC, MBC and MIC:MFC or MBC:MIC of the aqueous extract of *A. australe* (TAE), silver nitrate (AgNO_3_), silver nanoparticles (AgNPs), itraconazole (ITZ), penicillin (PEN) and gentamicin (GEN) against the microorganisms tested. All MIC, MFC and MBC data were expressed as μg lyophilized extract/mL for TAE and μg silver/mL for AgNO_3_ and AgNPs.

Species (N)	Compound	MIC-2		MIC-1		MIC-0		MFC | MBC		MFC:MIC | MBC:MIC	
		Range	Mode	Range	Mode	Range	Mode	Range	Mode	Range	Mode
*M. canis* (33)	TAE	> 1024	ND	> 1024	ND	> 1024	ND	ND	ND	ND	ND
	AgNO_3_	2–32	8	4–64	16	16–512	128	32–1024	512	2–8	4
	AgNPs	1–8	1	1–16	2	2–32	4	4–32	16	1–4	2
	ITZ	ND	ND	< 0.015–0.125	ND	ND	ND	ND	ND	ND	ND
*M. gypseum* (19)	TAE	> 1024	ND	> 1024	ND	> 1024	ND	ND	ND	ND	ND
	AgNO_3_	32–128	64	32–128	64	128–1024	512	512–1024	1024	2–8	4
	AgNPs	4–8	4	4–16	8	8–32	16	16–32	16	1–2	1
	ITZ	ND	ND	< 0.015–0.25	ND	ND	ND	ND	ND	ND	ND
*E. floccosum* (4)	TAE	> 1024	ND	> 1024	ND	> 1024	ND	ND	ND	ND	ND
	AgNO_3_	2–4	4	2–4	4	4–8	8	8–16	16	1–2	2
	AgNPs	1–4	1	1–4	2	2–4	4	4–8	8	1–2	2
	ITZ	ND	ND	< 0.015–0.03	ND	ND	ND	ND	ND	ND	ND
*T. rubrum* (31)	TAE	> 1024	ND	> 1024	ND	> 1024	ND	ND	ND	ND	ND
	AgNO_3_	4–32	16	4–64	16	16–128	32	32–512	64	2–4	4
	AgNPs	0.25–4	2	0.5–4	2	0.5–4	4	1–16	16	1–4	4
	ITZ	ND	ND	< 0.015–0.5	ND	ND	ND	ND	ND	ND	ND
*T. mentagrophytes* (22)	TAE	> 1024	ND	> 1024	ND	> 1024	ND	ND	ND	ND	ND
	AgNO_3_	8–32	16	8–32	32	8–128	32	32–256	64	2–4	4
	AgNPs	4–8	8	4–16	8	8–6	16	16–32	16	1–2	2
	ITZ	ND	ND	< 0.015–1	ND	ND	ND	ND	ND	ND	ND
*T. tonsurans* (6)	TAE	> 1024	ND	> 1024	ND	> 1024	ND	ND	ND	ND	ND
	AgNO_3_	4–16	8	4–32	8	8–32	16	16–64	32	2–4	2
	AgNPs	0.25–2	2	0.5–4	2	1–4	4	4–8	4	2–4	2
	ITZ	ND	ND	< 0.015–0.125	ND	ND	ND	ND	ND	ND	ND
*M. furfur* (40)	TAE	> 1024	ND	> 1024	ND	> 1024	ND	ND	ND	ND	ND
	AgNO_3_	1–8	4	1–8	4	2–8	8	4–32	16	2–4	2
	AgNPs	0.06–4	0.125	0.125–4	0.125	0.125–4	0.25	0.25–8	0.5	1–4	2
	ITZ	0.03–0.125	0.03	ND	ND	ND	ND	ND	ND	ND	ND
*M. furfur* CBS 7019	ITZ	ND	0.03	ND	ND	ND	ND	ND	ND	ND	ND
*M. sympodialis* (25)	TAE	> 1024	ND	> 1024	ND	> 1024	ND	ND	ND	ND	ND
	AgNO_3_	0.25–4	2	0.5–4	2	1–4	4	2–16	8	2–4	2
	AgNPs	0.015–2	0.125	0.03–2	0.125	0.03–4	0.25	0.06–4	0.25	1–4	2
	ITZ	0.015–0.06	0.03	ND	ND	ND	ND	ND	ND	ND	ND
*M. sympodialis *CBS 7222	ITZ	ND	0.03	ND	ND	ND	ND	ND	ND	ND	ND
*M. globosa* (9)	TAE	> 1024	ND	> 1024	ND	> 1024	ND	ND	ND	ND	ND
	AgNO_3_	0.25–1	0.5	0.25–2	0.5	0.5–4	2	1–16	8	2–4	2
	AgNPs	0.06–0.25	0.06	0.06–0.25	0.125	0.125–0.5	0.125	0.125–1	0.125	1–4	2
	ITZ	0.015–0.06	0.03	ND	ND	ND	ND	ND	ND	ND	ND
*M. globosa* CBS 7986	ITZ	ND	0.015	ND	ND	ND	ND	ND	ND	ND	ND
*M. restricta* (1)	TAE	> 1024	ND	> 1024	ND	> 1024	ND	ND	ND	ND	ND
	AgNO_3_	ND	0.5	ND	1	ND	2	ND	4	ND	2
	AgNPs	ND	0.25	ND	0.5	ND	1	ND	1	ND	1
	ITZ	ND	0.03	ND	ND	ND	ND	ND	ND	ND	ND
*C. albicans* (24)	TAE	> 1024	ND	> 1024	ND	> 1024	ND	ND	ND	ND	ND
	AgNO_3_	1–16	8	1–32	16	2–32	32	4–128	64	2–4	4
	AgNPs	0.25–8	0.5	0.25–16	2	0.5–16	4	2–32	8	2–4	4
	ITZ	< 0.015–0.125	ND	ND	ND	ND	ND	ND	ND	ND	ND
*C. albicans *ATCC 90028	ITZ	ND	0.125	ND	ND	ND	ND	ND	ND	ND	ND
*C. krusei* (21)	TAE	> 1024	ND	> 1024	ND	> 1024	ND	ND	ND	ND	ND
	AgNO_3_	1–16	8	1–32	16	2–32	32	4–128	64	2–4	4
	AgNPs	0.25–4	0.5	0.25–8	1	0.5–16	4	2–32	8	2–4	4
	ITZ	< 0.015–0.5	ND	ND	ND	ND	ND	ND	ND	ND	ND
ATCC 6258	ITZ	ND	0.5	ND	ND	ND	ND	ND	ND	ND	ND
*C. tropicalis* (16)	TAE	> 1024	ND	> 1024	ND	> 1024	ND	ND	ND	ND	ND
	AgNO_3_	1–8	8	1–16	8	2–32	16	4–64	32	2–4	4
	AgNPs	0.25–4	0.5	0.5–8	1	1–16	4	2–32	8	2–4	2
	ITZ	< 0.015–0.125	ND	ND	ND	ND	ND	ND	ND	ND	ND
*C. tropicalis* ATCC 750	ITZ	ND	0.125	ND	ND	ND	ND	ND	ND	ND	ND
*C. parapsilosis* (11)	TAE	> 1024	ND	> 1024	ND	> 1024	ND	ND	ND	ND	ND
	AgNO_3_	1–128	16	2–128	32	2–128	32	4–256	64	2–4	2
	AgNPs	0.5–4	2	1–8	4	1–16	8	2–32	16	2–4	2
	ITZ	< 0.015–0.125	ND	ND	ND	ND	ND	ND	ND	ND	ND
*C. glabrata* (6)	TAE	> 1024	ND	> 1024	ND	> 1024	ND	ND	ND	ND	ND
	AgNO_3_	1–16	4	1–32	16	2–32	16	4–128	64	2–4	4
	AgNPs	0.25–4	1	0.5–8	2	1–8	4	2–32	16	2–4	4
	ITZ	0.015–0.5	0.03	ND	ND	ND	ND	ND	ND	ND	ND
*C. glabrata* ATCC 2001	ITZ	ND	0.5	ND	ND	ND	ND	ND	ND	ND	ND
*S. aureus* (14)	TAE	> 1024	ND	> 1024	ND	> 1024	ND	ND	ND	ND	ND
	AgNO_3_	8–64	32	> 1024	ND	> 1024	ND	ND	ND	ND	ND
	AgNPs	0.5–4	1	1–8	4	2–16	8	8–64	32	4–8	4
	GEN	ND	ND	ND	ND	0.25–4	0.5	ND	ND	ND	ND
*S. aureus* ATCC 25923	GEN	ND	ND	ND	ND	ND	0.25	ND	ND	ND	ND
*P. aeruginosa* (8)	TAE	> 1024	ND	> 1024	ND	> 1024	ND	ND	ND	ND	ND
	AgNO_3_	4–64	16	> 1024	ND	> 1024	ND	ND	ND	ND	ND
	AgNPs	0.5–2	1	1–4	2	1–4	2	4–32	8	4–8	4
	GEN	ND	ND	ND	ND	0.125–2	0.5	ND	ND	ND	ND
*S. pyogenes* (8)	TAE	> 1024	ND	> 1024	ND	> 1024	ND	ND	ND	ND	ND
	AgNO_3_	8–32	16	> 1024	ND	> 1024	ND	ND	ND	ND	ND
	AgNPs	0.015–0.5	0.125	0.015–0.5	0.125	0.015–1	0.25	0.06–2	0.5	1–4	2
	PEN	ND	ND	ND	ND	< 0.015–0.03	ND	ND	ND	ND	ND

ND: Not determined.

MIC-2: the lowest concentration capable of inhibiting ≥ 50% of microbial growth compared to the growth control.

MIC-1: the lowest concentration capable of inhibiting ≥ 80% of microbial growth compared to the growth control.

MIC-0: the lowest concentration capable of inhibiting 100% of microbial growth.

Compounds with MIC values > 1024 μg/mL were considered inactive.

A compound was considered bactericidal or fungicidal if the ratio (MFC:MIC or MBC:MIC) was ≤ 4, and bacteriostatic or fungistatic if the ratio was > 4.

The values obtained with the control drugs were similar to those obtained by other authors^27,36–39^, which allowed us to confirm that we were working under standardized conditions.

## Discussion

In recent years, the World Health Organization (WHO) has emphasized the importance of research and development of new antimicrobial drugs as morbidity and healthcare costs have increased due to the rise of resistant strains^40^. In response, nanoparticles and medicinal plants have emerged as promising alternatives for the treatment of skin infections^3,8,10,11,16,17,41^. In addition, the development of more efficient green synthesis methods has become an important focus of researchers. Biological synthesis of silver nanoparticles using plants has proven to be cost-efficient and environmentally friendly and is a valuable alternative for large-scale production^10,13,41^. Ayurveda, the traditional Indian medicine, was perhaps the first to use metallic herbal preparations. These preparations, often called bhasmas, are used to treat many diseases. Silver bhasma is a medicine known since the seventh century BC, obtained after exposure of metallic silver through a series of physico-chemical processes in the presence of medicinal herbs. Today, bhasmas are claimed to be biologically produced nanoparticles^42^.

### Aqueous extract of *A. australe* (TAE)

The selection of plant material for this study was based on the ancestral knowledge of indigenous people in northeastern Argentina about medicinal plants for the treatment of skin infections^14,43^. TAE was used as both reducing and capping agent for AgNP synthesis. The obtained yield of 12% from TAE was lower than the values of 17.1%, 22.8% and 28.5% obtained by other authors^44–46^. Several factors influence this performance, including the plant material used and the extraction methodology, but also the place and time of collection, the chemical nature of the metabolites present in the plant, and their affinity for the solvent^47^. It has been shown that growth conditions lead to changes in plant composition, which translates into changes in extraction yield^17^.

On the other hand, chemical analysis of TAE revealed the presence of phenolic compounds, mainly flavonoids. Similar to other reports, the presence of these compounds was also detected in tapekué extracts, which are associated with antimicrobial properties and are responsible for the reducing property of the extract^48–50^. Likewise, it is known that the flavonoids contained in the aqueous plant extracts are mainly responsible for the reduction of silver ions and the formation of stable nanoparticles^13,21,41^.

### Silver nanoparticles (AgNPs)

In this work, the ionized chemical groups present in the extract allowed the rapid formation of AgNPs when TAE was mixed with AgNO_3_ solution at pH 9.5. The color change indicated the presence of AgNPs in the solution.

TEM images were used to determine the size of the metallic core and DLS to determine the hydrodynamic size, i.e., the size of the nanoparticle in solution including the metallic core, the coating phytochemicals, and the solvent layer. TEM images confirmed the presence of AgNPs with sizes and shapes similar to those obtained by other authors with other plants^41^. DLS analysis revealed a monomodal population. Moreover, absolute values of zeta potential higher than 30 mV indicate stable nanoparticles, which could be related to the electrostatic repulsion of the anions present in the colloidal suspension^51^. These results explain the good stability of the synthesized AgNPs, which remained stable as colloids for more than 6 months.

EDX analysis verified the purity of the AgNPs and the presence of traces of other elements that could have originated from the biomolecules of TAE that were bound to the surface of the nanoparticles. FTIR analysis allowed us to identify the functional groups responsible for the reduction of silver ions. It revealed peaks at 1636, 2100 and 3300 cm^-1^ corresponding to the C = O functional groups of the amide (1640 cm^-1^), the C≡C stretch (2100 cm^-1^) and the N–H/O–H vibrational stretch of the amine (3370 cm^-1^), which are similar to the functional groups of AgNPs synthesized with quercetin^21^. In addition, other authors have shown that quercetin is present in tapekué extracts^48,52^. The results obtained in this work suggest that quercetin could be the main metabolite responsible for the reduction of silver ions to AgNPs, according to Jain et al.^21^. Moreover, the obtained value of negative zeta potential could be due to the hydroxyl groups of quercetin present on the surface of the nanoparticles, generating a negative charge on these particles.

Quercetin (3,3′,4′,5,7-pentahydroxylflavone) is a typical phenolic compound. It is a flavonoid widely distributed in the plant kingdom and one of the most important compounds in some plants, exhibiting anti-inflammatory, anti-allergic and protective properties against various diseases associated with oxidative stress. In recent years, this flavonoid has attracted much attention, especially as an antioxidant agent^53^.

### Antioxidant activity

In this work, TAE showed significant antioxidant activity. In contrast, AgNO_3_ solution showed no activity, while AgNPs showed lower antioxidant activity (55%) than the TAE in both assays (DPPH and FRAP). This resulted in the inability to determine the EC_50_ for AgNP and AgNO_3_ solutions. The activity shown by AgNPs could be due to the phenolic compounds of TAE present on the surface of the nanoparticles, which act as antioxidants and capping agents.

The antioxidant property of a plant is given mainly by the composition and concentration of phenolic compounds and to a lesser extent by other components. Phenolic compounds are involved in the stabilization of free radicals and metals by acting as electron donors. The antioxidant activity of many polyphenols results essentially from the ease with which the hydroxyl group is donated to a free radical and the ability of the aromatic structure to carry an unpaired electron^54^. The results show that TAE is rich in phenolic compounds, which justifies its higher antioxidant activity. Therefore, TAE polyphenols played an important role both as silver ion reducing agents and AgNP capping agents.

### Cytotoxic activity

The cytotoxicity of TAE, AgNO_3_ and AgNP solutions was studied on PBMCs. PBMCs are cells of the immune system consisting of lymphocytes (T cells, B cells, NK cells) and monocytes. Cytotoxicity on PBMCs represents the potential for immune responses and immunotoxicity and allows prediction of immune effects during preclinical safety evaluation. It also helps to understand the interactions between nanomaterials and cells of the immune system^55^.

Cytotoxicity assays of the components used for the synthesis of the nanoparticles showed that AgNO_3_ was cytotoxic, while TAE was not cytotoxic to PBMCs at the concentrations evaluated. Moreover, the plant extract induced low cell proliferation compared to untreated cells. It has been reported that metabolites present in plants, mainly phenolic compounds, affect the nonspecific immune response by mainly increasing phagocytosis and proliferation of macrophages and neutrophils^56^. The non-toxicity property shown by TAE is of utmost importance in the context of AgNP production for medicinal applications, especially as a potential topical treatment for skin infections.

On the other hand, AgNP solution showed variations in cell viability as a function of dose and time. Cell viability reached values below 70% only at AgNP concentrations ≥ 16 µg/mL after 24 h of incubation, but was above 70% after 72 h of incubation, indicating the non-cytotoxicity of the synthesized nanoparticles at the tested concentrations^25^.

Since AgNO_3_ was cytotoxic, while AgNPs and TAE were found to be non-cytotoxic and even TAE showed a slight proliferative effect, we can conclude that the reduction in cell viability shown by the synthesized AgNPs is related to the silver content, but TAE metabolites present on the surface of the nanoparticles reduce the cytotoxic effect and modulate the immune response. Therefore, the use of silver in humans may have toxic effects, but these effects can be attenuated when silver is incorporated as coated nanoparticles. In other words, a certain amount of silver in the form of silver ions is more toxic than the same amount in the form of nanoparticles coated with phytocompounds.

Our results are in agreement with those of Orta-García et al.^57^ who showed that uncoated AgNPs induce significant cytotoxic effects on PBMCs at relatively low concentrations (< 5 μg/mL) and short exposure times (3 to 12 h), suggesting that the coating reduces the area of the active surface of AgNPs and thus the sites of interaction with cellular components.

### Antimicrobial activity

The antimicrobial activity of AgNPs, AgNO_3_ and TAE, was tested against 298 fungi and bacteria causing SSTIs. Three endpoint values (MIC-0, MIC-1, and MIC-2) were used along with the determination of CFM, as there is still no consensus on which is the best predictor of the in vivo behavior of these compounds.

Although tapekué is used in traditional medicine to treat skin infections, TAE was inactive against the microorganisms tested in this work. However, considering the possibility of poor water solubility of the active compound, we cannot claim that the plant lacks this well-known traditional property. This position is in agreement with the results of Portillo et al.^45^, who showed that tapekué extracts obtained with organic solvents had higher antimicrobial activity than the aqueous extract.

**Figure 3:**
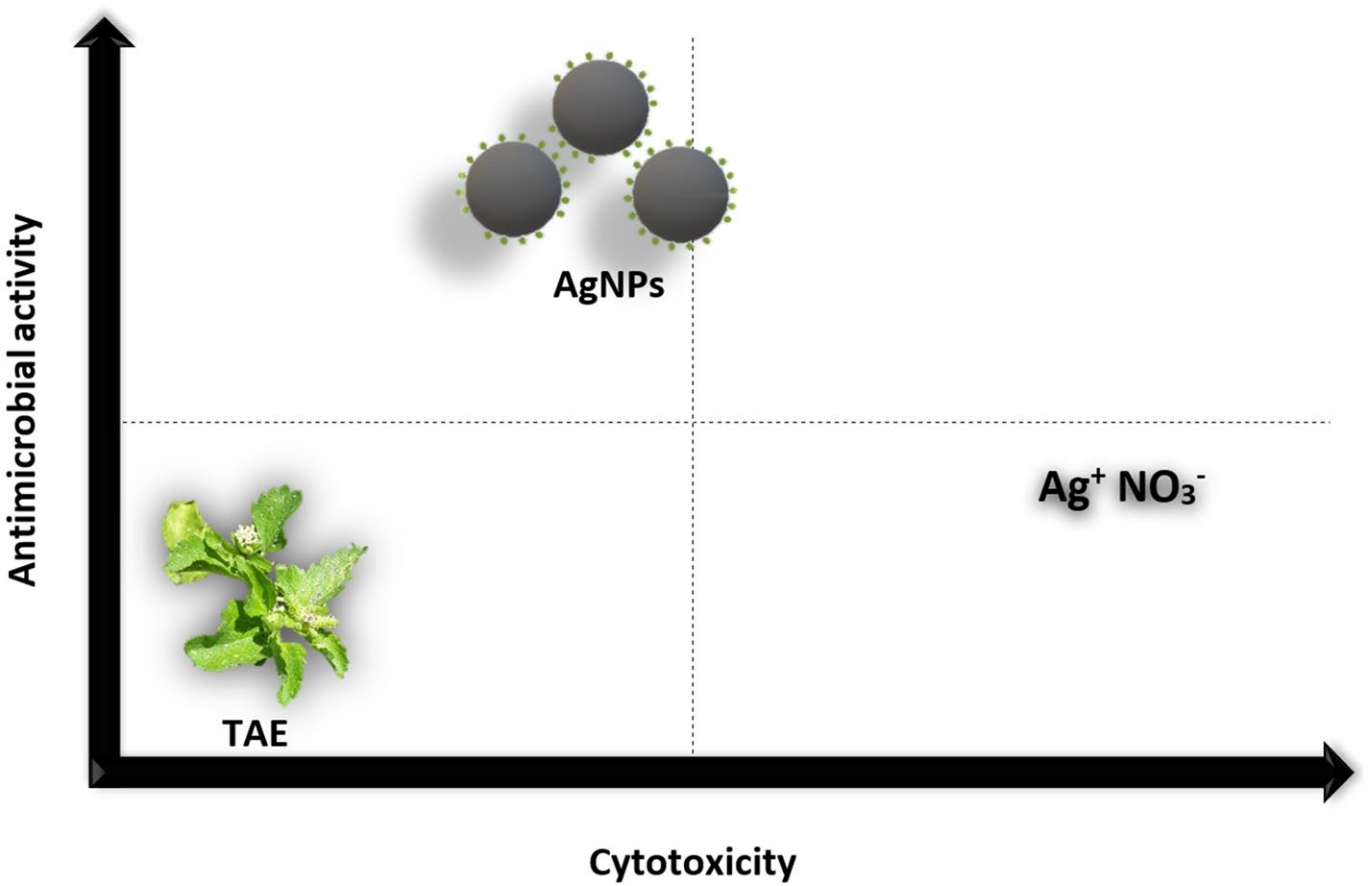
Graphical representation of antimicrobial and cytotoxic activity of TAE, AgNO_3_ and AgNPs. Only the synthesized AgNPs showed high antimicrobial activity with moderate cytotoxicity compared to TAE and AgNO_3_.

AgNO_3_ showed antimicrobial activity but with higher MIC and CFM values than those of the synthesized nanoparticles, and its toxicity precludes it as an antimicrobial agent. Therefore, AgNP solution was a better antimicrobial agent than the components used for its synthesis (Fig. 3).

Variations in MIC and CFM values were observed between isolates of the same species and between species of the same genus. These variations highlight the importance of testing multiple isolates in the evaluation of antimicrobial activity.

According to the MFC values, the tested *S. pyogenes* strains and *Malassezia* species were most inhibited by AgNPs. *M. globosa*, the main causative agent of pityriasis versicolor and seborrheic dermatitis^5^, was the most susceptible microorganism to the synthesized nanoparticles. Moreover, methicillin-resistant *S. aureus* strains were inhibited by AgNPs. This indicates the potential use of AgNPs as antimicrobial agents against fungi and bacteria.

The values of MFC:MIC ratio obtained for AgNPs against the tested fungi were consistent with fungicidal activity. However, a different behavior was obtained against bacteria. According to the mode values obtained for MBC:MIC ratios, AgNPs showed bactericidal activity, but some strains of *S. aureus* and *P. aeruginosa* showed values ≥ 4 associated with bacteriostatic activity.

MIC-0 and MFC values greater than 1024 μg/mL were obtained for AgNO_3_ against bacteria; therefore, the MFC:MIC ratio could not be determined. However, the MIC-2 values were determined. In addition, sustained growth was observed at AgNO_3_ concentrations above the MIC-2 (trailing effect). This effect occurs with drugs that have bacteriostatic activity and is not normally observed with bactericidal drugs. Although AgNO_3_ may have a bacteriostatic effect, we did not consider AgNO_3_ itself as an antimicrobial agent because of its toxicity.

The use of less stringent endpoint readings, such as MIC-2 and MIC-1, is associated with drugs with fungistatic/bacteriostatic activity, whereas MIC-0 is generally associated with drugs with biocidal activity^28,29^. Consequently, MIC-0 could be considered as the best endpoint reading for AgNPs against the tested fungi and bacteria.

To confirm fungicidal or bactericidal activity, further studies, such as time-kill assays, are needed to correlate these results. In addition, it is important to note that the choice of endpoint reading for a clinical drug is based on the distribution of their MIC, pharmacokinetic and pharmacodynamic parameters, animal models, and therapeutic outcomes^58^.

The reasons for the excellent antimicrobial activity of AgNPs are not yet clear. However, it is known that the mode of action of AgNPs is the result of several simultaneous processes by which AgNPs can circumvent most microbiological resistance mechanisms that occur with classical antimicrobial agents. Therefore, new strategies against multidrug-resistant microorganisms are increasingly based on the search for synergistic effects between antimicrobial agents of clinical use and metallic nanoparticles^16,59^.

## Conclusion

In summary, green synthesis using TAE proved to be a simple, environmentally friendly and economical method for the preparation of AgNPs with improved properties compared to their synthesis components.

AgNPs were not cytotoxic at the concentrations required to inhibit microbial growth, whereas AgNO_3_ was cytotoxic. AgNPs were fungicidal and bactericidal, while AgNO_3_ was fungicidal but bacteriostatic. TAE was inactive against the main microorganisms causing SSTIs and not cytotoxic to PBMCs.

The antimicrobial activity of AgNPs is due to the silver and not to the phytocompounds present on the surface of the nanoparticles. TAE plays a more important role in the formation of stable AgNPs and possibly as a modulator of cytotoxic and immune response.

The combination of traditional medicine with nanotechnology gives us a great opportunity to develop new antimicrobial agents. This work enabled us to rediscover a technology formerly known as bhasmas for the treatment and prevention of SSTIs.

## Supplementary Information

Supplementary Information.
