# Supplementary material for: Antimicrobial and cytotoxic activity of green synthesis silver nanoparticles targeting skin and soft tissue infectious agents
Source: Sci Rep. 2021 Jul 15;11:14566. doi: 10.1038/s41598-021-94012-y (PMC8282796; doi:10.1038/s41598-021-94012-y)
Supplement: Supplementary file 1 — Supplementary Information. [file 41598_2021_94012_MOESM1_ESM.doc]

**SUPPLEMENTARY FILE**

**Antimicrobial and cytotoxic activity of green synthesis silver nanoparticles targeting skin and soft tissue infectious agents**

Javier Mussin, Viviana Robles-Botero, Rocío Casañas-Pimentel, Florencia Rojas, Letizia Angiolella, Eduardo San Martín-Martínez, Gustavo Giusiano.

**Materials and methods**

**Total phenolic content**

The total phenolic content of TAE was analyzed spectrophotometrically using the Folin-Ciocalteu method^18^, with some modifications.

The lyophilized extract (LE) was reconstituted with sterile deionized water to give a concentration of 1 mg LE /mL. Then, 20 μL of TAE (1 mg LE/mL) was added to 1.4 mL of distilled water, followed by 100 μL of Folin-Ciocalteau reagent (2N). The solution was allowed to stand at room temperature for 3 min. Then 300 μL of a sodium carbonate solution (20% m/v) and 180 μL of distilled water were added. The solution was allowed to stand in the dark for 100 min and the absorbance was determined at a wavelength of 760 nm using a UV-visible spectrophotometer (Multiskan Go, Thermo Fisher, Finland). The results are expressed as the mean gallic acid equivalent mass in mg per g of lyophilized extract (mg GAE/g LE) ± standard deviation (SD).

**Total flavonoid content**

It was determined for TAE according to Dewanto et al. ^19^ with modifications. 200 μL of TAE (1 mg LE /mL) was added to 1000 μL distilled water containing 60 μL NaNO_2_ solution (5%). After 6 min, 120 μL AlCl_3_·6H_2_O solution (10%) was added and allowed to stand for 5 min. Then 400 μL NaOH (1M) and 220 μL distilled water were added and stirred. Immediately, the absorbance at 510 nm was read. Total flavonoid content was expressed as the mean quercetin equivalent mass in mg per g of lyophilized extract (mg QE/g LE) ± SD.

**Green synthesis of silver nanoparticles**

In order to obtain small and stable nanoparticles, the green synthesis was carried out according to the results obtained by other authors^12,20,21^, with some modifications. TAE (50 mg LE/mL) was adjusted to pH 9.5 with a 0.1 N NaOH solution. Then, 10 mL of TAE was added to 190 mL of a 1 mM AgNO_3_ solution to reduce silver ions (Ag^+^). The reaction was carried out at 95 °C for 15 min. The AgNP solution was purified by centrifugation and redispersion (3 times) of the pellet in sterile deionized water at 15,000 rpm for 20 min to get rid of all uncoordinated biological molecules.

**Ferric reducing antioxidant power (FRAP) assay**

The antioxidant activity of TAE, AgNO_3_ and AgNPs was determined using the FRAP assay according to Quiroz-Reyes et al^22^. This method is based on the reduction of Fe^3+^ ions by the sample and the determination of the colored complex of Fe^2+^ with TPTZ (2,4,6-tri(2-pyridyl)-1,3,5-triazine) at 595 nm. The reagent FRAP was prepared by mixing 25 mL of a 0.3 M acetate buffer (pH 3.6), 2.5 mL of a TPTZ solution (0.01 M) and 2.5 mL of a solution of FeCl_3_·6H_2_O (0.02 M) at 37 °C. 100 µL of the sample (1 mg lyophilized extract of TAE /mL; 1 mM AgNO_3_; or the obtained AgNP solution) was mixed with 1900 μL of the solution FRAP and left in the dark for 30 min. The absorbance was read at a wavelength of 593 nm. Each sample was analyzed in triplicate. The results are expressed as the mean Trolox equivalent mass in μmol per g of lyophilized extract (μmol TE/g LE) ± SD.

**DPPH radical scavenging capacity**

The antiradical capacity was determined using the DPPH (2,2-diphenyl-1-picrylhydrazyl) assay according to the methodology proposed by Molyneux^23^, with some modifications. 500 μL of sample (1 mg LE/mL; 1 mM AgNO_3_; or obtained AgNP solution) was mixed with 125 μL Tris-HCl buffer (pH 7.4, 0.1M). To this solution, 500 μL DPPH (431.1 μM) was added. After resting for 30 min in the dark, the absorbance at 517 nm was determined. The effective concentration 50% (EC_50_), defined as the amount of antioxidant required to reduce the initial concentration of DPPH radical by 50%, was calculated. The results are expressed in µg/mL. In addition, the percentage of DPPH reduction was calculated using the following equation:


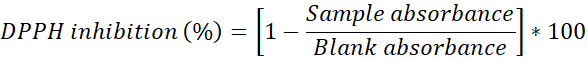


**Cytotoxicity assay**

The cytotoxicity of TAE, AgNO_3_ and AgNPs was determined against peripheral blood mononuclear cells (PBMCs) as previously described^24^, with modifications.
PBMCs were obtained from leukocyte packages from healthy donors (Blood Bank, Central Military Hospital, Mexico City) using the density gradient medium Lymphoprep (Alere Technologies AS, Norway) according to the manufacturer's instructions. PBMCs were seeded in 96-well plates at a confluence of 50,000 cells per well in a volume of 50 μL culture medium. The cytotoxicity of TAE, AgNO_3_, and AgNPs on these cells was tested by adding 50 μL of X- VIVO medium (Lonza, USA) to each well, which contained different concentrations of each compound (100 μL final volume). The final concentrations of the compounds were 2, 4, 8, 16, and 32 μg/mL (concentrations are expressed as μg LE/mL culture medium for TAE and in terms of silver content for AgNPs and AgNO_3_). Cells were incubated for 24 and 72 h at 37 °C in a humidified atmosphere with 5% CO_2_. Untreated cells were considered as negative control and paclitaxel 2.5 µg/mL (Sigma Aldrich, USA) as positive control.

After incubation, 10 μL of WST-1 reagent solution (Clontech, USA) was added to each well and incubated again for 90 min at 37 °C in a humidified atmosphere with 5% CO_2_. The plates were then shaken for 5 sec, and absorbance was determined at 450 nm using an ELISA instrument (Multiskan Go, Thermo Scientific). The different samples were compared with the negative control and the percent cell viability was calculated. Samples with viability percentages below 70% were considered cytotoxic according to the recommendations of ISO 10993-5^25^.

**Minimum fungicidal concentration (MFC) and minimum bactericidal concentration (MBC)**

The MFC and MBC of TAE, AgNO_3_ and AgNPs were determined according to the procedures established by other authors^30–33^, with modifications.
After reading the MIC, the contents of each well with complete growth inhibition were homogenized using a micropipette and the entire volume (200 µL) of these wells were subcultured onto plates containing Sabouraud dextrose agar for *Candida* spp., modified Dixon agar for *Malassezia* spp., Lactrimel agar for dermatophytes, blood agar for *S. pyogenes* and Mueller-Hinton agar for *S. aureus* and *P. aeruginosa*. Aliquots were plated on the agar and after drying, the plate was streaked. The plates were incubated at 35 °C for 48 h for *Candida* spp. and bacteria, at 32 °C for 72 h for *Malassezia* spp. and at 28 °C for 7 days for dermatophytes. The MBC and MFC were defined as the lowest TAE, AgNO_3_ or AgNP concentrations at which no colonies were observed.
